# *Calamagrostis arundinacea* Extract Mitigates Testosterone Induced Prostatic Hyperplasia in Rats

**DOI:** 10.3390/ph19030453

**Published:** 2026-03-11

**Authors:** Poornima Kumbukgahadeniya, Eun-Bok Baek, Seung-Hoon Lee, Dae-In Ha, Eun-Ju Hong, Jun-Yeop Song, Won-Kee Yoon, Hyo-Jung Kwun

**Affiliations:** 1 Department of Veterinary Pathology, College of Veterinary Medicine, Chungnam National University, Daejeon 34134, Republic of Korea; plakshinikg@o.cnu.ac.kr (P.K.); baekeunbok@hanmail.net (E.-B.B.); lsh@cnu.ac.kr (S.-H.L.); madaein@gmail.com (D.-I.H.); bioeun59@naver.com (E.-J.H.); song1038@o.cnu.ac.kr (J.-Y.S.); 2 Department of Physiology, College of Medicine, Jeju National University, Jeju-si 63243, Republic of Korea; 3 Laboratory Animal Resource Center, Korea Research Institute of Bioscience and Biotechnology, Cheongju-si 28116, Republic of Korea

**Keywords:** *Calamagrostis arundinacea*, benign prostatic hyperplasia, apoptosis, PI3K/Akt mTOR signaling

## Abstract

**Background**: Benign prostatic hyperplasia (BPH) is an age-associated urological condition defined by abnormal multiplication of both stromal and epithelial components within the prostate. *Calamagrostis arundinacea* (CA), a species of perennial grass native to East Asia, has been recognized for its anti-inflammatory and antioxidant biological activities. The present study examined whether CA extract could attenuate prostatic enlargement induced by testosterone propionate (TP) in rats. **Methodology**: To establish the experimental model, rats received subcutaneous TP injections (3 mg/kg/day) for four consecutive weeks. During the same period, an extract of CA (150 mg/kg/day) was orally administered. **Results**: TP-treated animals developed significant prostatic enlargement, whereas CA supplementation markedly reduced prostate weight and significantly decreased circulating dihydrotestosterone (DHT) and testosterone levels. Microscopic analysis demonstrated that CA mitigated glandular epithelial thickening and suppressed hyperplastic alterations. In addition, CA reduced proliferating cell nuclear antigen (PCNA) expression and increased apoptotic cell numbers, as evidenced by TUNEL staining. Gene expression analysis further revealed significant downregulation of insulin-like growth factor-2 (*Igf-2*), transforming growth factor-β (*Tgf-β*), and vascular endothelial growth factor (*Vegf*), in CA-treated prostates. Moreover, CA inhibited activation of the PI3K/Akt/mTOR signaling cascades by reducing phosphorylation of Akt and mTOR. **Conclusions**: Overall, these results indicate that CA extract alleviates testosterone-induced BPH through suppression of growth-related signaling cascades and induction of apoptosis, suggesting its potent value as a phytotherapeutic strategy for BPH management.

## 1. Introduction

Among the aging male population, benign prostatic hyperplasia (BPH) represents a frequent urological challenge. Clinical data suggest its incidence escalates significantly with age, affecting nearly half of men in their sixties and reaching up to 80% in those over 90 years old [1,2,3]. BPH is marked by a proliferative, benign condition and progressive histopathological shifts within the transitional zone of the prostate, marked by the development of prostatic nodules, inflammatory response, tissue fibrosis, and changes in smooth muscle tone. These pathological alterations contribute to urethral obstruction and the precipitating onset of lower urinary tract symptoms (LUTSs) [1,2,3,4]. The worldwide burden of BPH is anticipated to increase significantly as life expectancy increases, highlighting the need for diverse therapeutic options [4,5]. Although the precise molecular mechanisms underlying BPH remain unclear, the condition is believed to result from a disproportion between cellular proliferation and programmed cell death in both the epithelial and stromal compartments [6,7,8]. Normal prostate growth and function are based upon the transformation of testosterone to dihydrotestosterone (DHT), result binds to receptors of androgen (ARs) to stimulate prostate cell proliferation and protein synthesis [4,9,10]. The dysregulation of this androgenic signaling remains a primary focus of research, as it directly governs the expression of genes associated with cellular growth [11,12]. With aging, serum testosterone levels decline; however, patients with BPH often exhibit elevated intraprostatic DHT levels, which are positively correlated with prostate enlargement [9]. In addition, various growth factors, including transforming growth factor-beta (TGF-β) family members, vascular endothelial growth factors (VEGFs), and insulin-like growth factors (IGFs), have been involved in the pathogenesis of BPH [13,14,15]. These mediators facilitate stromal epithelial interactions within the prostate to modulate cellular proliferation, differentiation, and apoptosis [16,17].

Management of symptomatic BPH encompasses lifestyle modifications, pharmacological interventions, minimally invasive device-based therapies, and surgical procedures [18,19]. The pharmacological treatments include α1-adrenergic receptor antagonists (α1-blockers), 5α-reductase inhibitors (5-ARIs), and phosphodiesterase type 5 (PDE5) inhibitors, given either as monotherapy or in combination [19,20,21]. Among these options, 5-ARIs, α1-blockers, and combination therapies such as tamsulosin plus dutasteride are currently the most prescribed regimens [19,20,21]. In our study, Finasteride, a competitive and specific inhibitor of Type II 5-AR, which reduces the conversion of testosterone to dihydrotestosterone (DHT), was utilized as a reference standard due to its valid clinical efficacy in treating BPH [22,23]. In addition, phytotherapy, involving the use of bioactive plant extracts, has emerged as a complementary and increasingly popular approach for BPH management [24,25,26,27]. Herbal or plant-derived therapeutic agents are widely utilized in Europe and have gained growing acceptance in other parts of the world [24,25,26,27]. Natural dietary polyphenols and flavonoids are being extensively investigated as potential alternatives to conventional drugs because of their versatile biological activities, such as anti-proliferative properties and anti-inflammatory [28].

*Calamagrostis arundinacea* (Poaceae) is a species of perennial grass that is distributed across regions of Europe and Asia [29,30]. Ethnobotanical surveys have recently reported its traditional medicinal use in rural communities, particularly in Turkey, where it has been applied for its anti-inflammatory properties [31]. Although detailed pharmacological and phytochemical studies on *C. arundinacea* remain limited, it is believed to contain phytochemical constituents such as phenolic acids and flavonoids, as seen in other species of the genus Calamagrostis [32]. These substances are known to possess antioxidant, antimicrobial, and anti-inflammatory properties, suggesting that *C. arundinacea* may hold therapeutic potential [33,34]. Moreover, associations of *C. arundinacea* with fungi, such as Claviceps purpurea, have been reported to produce ergot alkaloids, highlighting the plant’s biochemical diversity and its relevance to pharmacological research [34]. Thus, while *C. arundinacea* has a basis in traditional medicine, additional studies are needed to identify its active compounds and confirm its therapeutic efficacy.

To date, the protective potential of *C. arundinacea* (CA) against BPH has not been elucidated. Accordingly, this study sought to analyze the pathophysiology and molecular processes responsible for the therapeutic impact of CA in a testosterone-induced BPH rat model.

## 2. Results

### 2.1. Analysis of UPLC-QTOF-MS and Physicochemical Characterization of CA Extract

**Figure 1:**
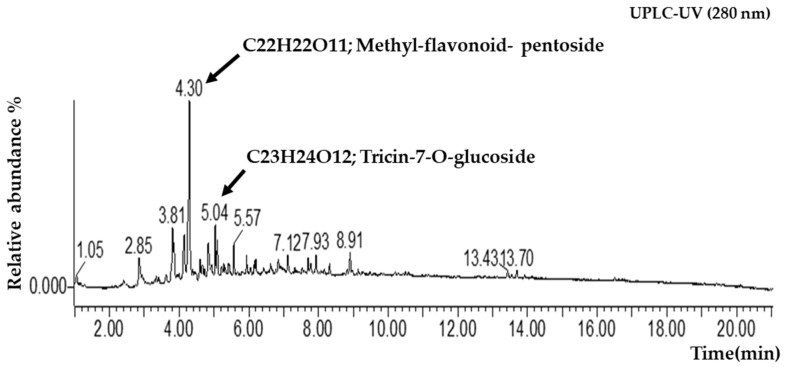
UPLC-UV and mass spectrometry (MS) analysis of CA extract. The UPLC-UV chromatogram obtained at 280 nm identified methyl-flavonoid-pentoside (RT 4.30 min) and tricin-7-O-glucoside (RT 5.04 min) as the major constituents.

Physicochemical characterization was performed using a combination of UPLC-QTOF-MS and spectroscopic methods. UPLC-UV analysis at 280 nm revealed two major components with high resolution. Peak 1 was identified as a Methyl-flavonoid-pentoside (C_22_H_21_O_11_), and Peak 2 was definitively identified as Tricin 7-O-glucoside (C_23_H_23_O_12_). The relative abundance of Methyl-flavonoid-pentoside and Tricin 7-O-glucoside is 45.2% and 32.8%, respectively (Figure 1). 

### 2.2. Cytotoxic Analysis of CA Extract

**Figure 2:**
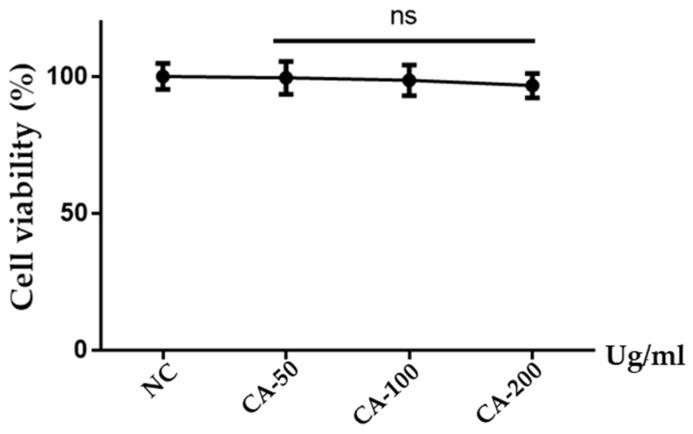
Cytotoxicity profile of CA extract in LNCaP cells. The viability of cells was measured using the MTT assay after 24 h of incubation at different concentrations of CA extract (50, 100, and 200 µg/mL). NC represents the vehicle-treated controls (0.1% DMSO). Horizontal lines above the bars indicate the groups compared in the statistical analysis. Results are reported as mean ± SD. Statistical analysis was conducted using one-way ANOVA, subsequently followed by Tukey’s post hoc test. ns, not-significant.

To evaluate the safety profile and potential cytotoxic effects of the Calamagrostis arundinacea (CA) extract, an MTT assay was performed on LNCaP human prostate cancer cells. According to the figure, cells were treated using different concentrations of CA extraction, such as 50, 100, and 200 µg/mL, for 24 h, which did not result in significant cytotoxicity (Figure 2). 

### 2.3. CA Extract Attenuates Prostate Weight in the Rat BPH Model

The prostate relative weight was determined by normalizing the prostate mass to the total body weight of each animal. As summarized in Table 1, animals in the group of BPH exhibited a marked increase in relative prostate weight (0.345% ± 0.076%) compared with the NC group (0.186% ± 0.032%). In contrast, administration of finasteride (0.215% ± 0.059%) or CA extract (0.286% ± 0.045%) attenuated this increase, resulting in markedly lower values than observed in the untreated BPH cohort. The calculated percentages of prostate growth suppression were 37.86% in the treated with finasteride (BPH + FIN) group and 17.34% in the CA-treated (BPH + CA) group.

### 2.4. CA Extract Reduces Serum Testosterone and Prostate DHT in Testosterone-Induced Rat BPH Model

**Figure 3:**
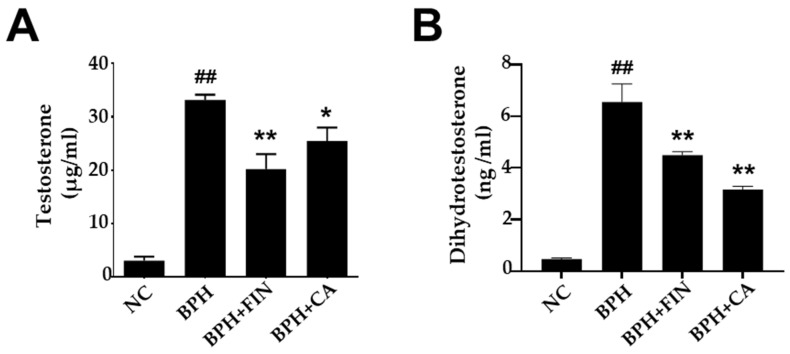
CA reduces serum testosterone and prostate DHT levels in the rat BPH model. (**A**) Serum levels of testosterone. (**B**) Levels of prostatic DHT. The NC group consisted of control rats receiving corn oil and PBS. The BPH group injected testosterone propionate (TP, 3 mg/kg) together with PBS. The FIN group received TP in combination with finasteride (10 mg/kg), and the CA group was injected with TP and CA extract (150 mg/kg). Results are reported as mean ± SD. Statistical analysis was conducted using one-way ANOVA, subsequently followed by Tukey’s post hoc test. Statistical significance was defined as ^##^ *p* < 0.01 compared with the NC group, and * *p* < 0.05 or ** *p* < 0.01 compared with the BPH group.

Serum testosterone concentrations were elevated in the BPH group relative to those observed in the NC group. In contrast, both finasteride and CA extract administration significantly decreased serum testosterone levels compared with the untreated BPH group (Figure 3A). Comparable patterns were observed in serum DHT levels (Figure 3B).

### 2.5. CA Extract Decreases Epithelial Thickness in the Prostate Gland Epithelium of the Testosterone-Induced Rat BPH Model

**Figure 4:**
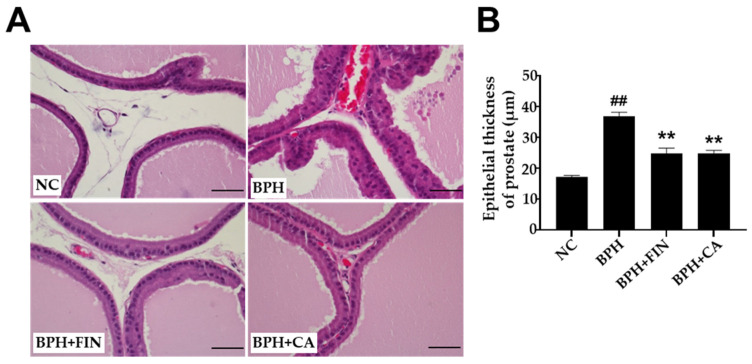
CA decreases the prostate epithelium thickness (**A**). Prostate tissues were stained with hematoxylin-eosin and observed under an optical microscope at ×200 magnification (scale bar 100 µm). (**B**) Mean epithelial thickness of prostate (mm). The NC group consisted of control rats receiving corn oil and PBS. The BPH group injected testosterone propionate (TP, 3 mg/kg) together with PBS. The FIN group received TP in combination with finasteride (10 mg/kg), and the CA group was injected with TP and CA extract (150 mg/kg). Sections were stained with H&E; purple indicates nuclei and pink indicates cytoplasm. Results are reported as mean ± SD. Statistical analysis was conducted using one-way ANOVA, subsequently followed by Tukey’s post hoc test. Statistical significance was defined as ^##^ *p* < 0.01 compared with the NC group, and ** *p* < 0.01 compared with the BPH group.

Sections obtained from the ventral prostates of the NC group and stained with hematoxylin-eosin did not exhibit any histological alteration in the lining of the epithelium. In contrast, samples from the BPH group displayed noticeable thickening in the lining of the epithelium and glandular hyperplasia when compared to the NC group (Figure 4A). The administration of finasteride and CA extracts to each respective group markedly reduced the thickening of the epithelium lining, compared to the BPH group (Figure 4B).

### 2.6. CA Extract Reduces Prostatic Cell Proliferation and Enhances Prostatic Cell Apoptosis in Testosterone-Induced Rat BPH Model

**Figure 5:**
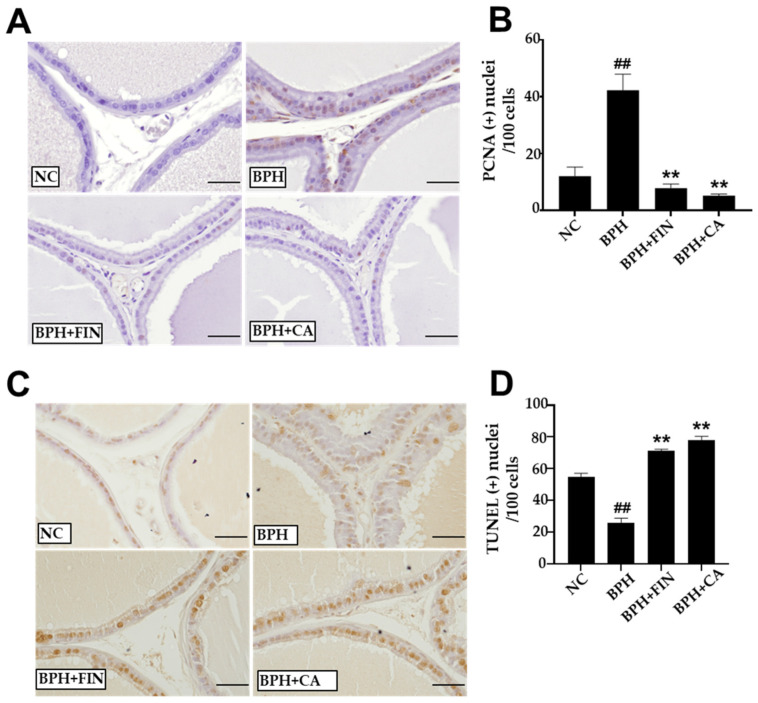
CA suppresses proliferation and promotes apoptosis among prostate cells in the rat BPH model. (**A**) Prostate epithelial cells stained with PCNA antibody (×400) (scale bar 50 µm). (**B**) The percentage of PCNA-positive cells; brown staining indicates PCNA-positive nuclei. (**C**) Prostate tissue was subjected to TUNEL staining; brown staining indicates TUNEL-positive cells (×400) (scale bar 50 µm). (**D**) The percentage of TUNEL- labeled cells. The NC group consisted of control rats receiving corn oil and PBS. The BPH group injected testosterone propionate (TP, 3 mg/kg) together with PBS. The FIN group received TP in combination with finasteride (10 mg/kg), and the CA group was injected with TP and CA extract (150 mg/kg). Data are represented as mean ± standard deviation (SD). Results are reported as mean ± SD. Statistical analysis was conducted using one-way ANOVA, subsequently followed by Tukey’s post hoc test. Statistical significance was defined as ^##^ *p* < 0.01 compared with the NC group, and ** *p* < 0.01 compared with the BPH group.

Immunohistochemical staining demonstrated a notable increase in the number of PCNA-positive cells in the BPH group (Figure 5A). Conversely, in the finasteride and CA groups, the number of PCNA-positive cells was markedly lower than that in the BPH group (Figure 5B). Compared to the NC group, there were markedly fewer terminal deoxynucleotidyl transferase dUTP nick-end labeling (TUNEL)-positive cells in the BPH group (Figure 5C). Moreover, in comparison to the BPH group, there was a significant increase in TUNEL-positive cells in both the finasteride and CA groups (Figure 5D).

### 2.7. CA Extract Impacts Growth Factors in Testosterone-Triggered Rat BPH Model

**Figure 6:**
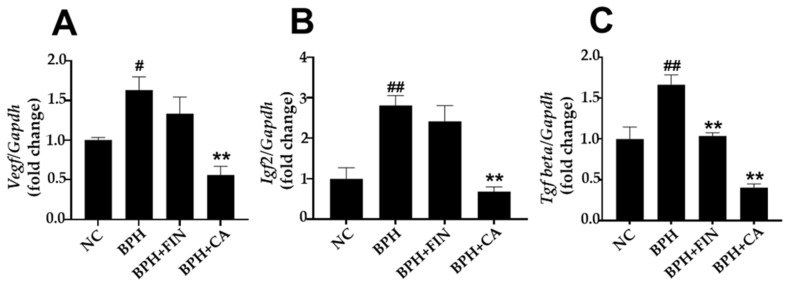
CA inhibits growth factors in the rat BPH model. The mRNA levels of (**A**) *Vegf*, (**B**) *Igf-2*, and (**C**) *Tgf-β* in prostate tissue. The fold change in target gene expression relative to the endogenous control (*Gapdh*) was calculated using the 2^−ΔΔCt^ method. *Gapdh* was used as the endogenous housekeeping control due to its stable expression levels across experimental groups. Relative mRNA expression levels were analyzed using the 2^−ΔΔCt^ method, which normalizes the target gene’s cycle threshold (Ct) to the endogenous control and compares it to the untreated control. The NC group consisted of control rats receiving corn oil and PBS. The BPH group injected testosterone propionate (TP, 3 mg/kg) together with PBS. The FIN group received TP in combination with finasteride (10 mg/kg), and the CA group was injected with TP and CA extract (150 mg/kg). Results are reported as mean ± SD. Statistical analysis was conducted using one-way ANOVA, subsequently followed by Tukey’s post hoc test. Statistical significance was defined as ^#^ *p* < 0.05 or ^##^ *p* < 0.01 compared with the NC group, and ** *p* < 0.01 compared with the BPH group.

To investigate the impact of CA administration on growth factor expression in the prostate tissue of rats, RT-PCR was performed. The results demonstrated that *Vegf* mRNA expression was significantly higher in the BPH cohort compared with the normal group. In contrast, the CA group shows a substantial reduction in *Vegf* mRNA expression compared to the BPH group (Figure 6A). The BPH group showed a marked increase in *Igf-2* mRNA levels compared to the normal group, whereas the CA group demonstrated a significant decrease in *Igf-2* mRNA levels compared to the BPH group (Figure 6B). Furthermore, the BPH group exhibited significantly higher relative mRNA expression of *Tgf-β* compared to the NC group, while the finasteride and CA cohorts exhibited a significant decrease in the relative mRNA levels of *Tgf-β* compared with the BPH group (Figure 6C).

### 2.8. CA Extract Reduces Akt and mTOR Pathway Activations in Testosterone-Induced Rat BPH Model

**Figure 7:**
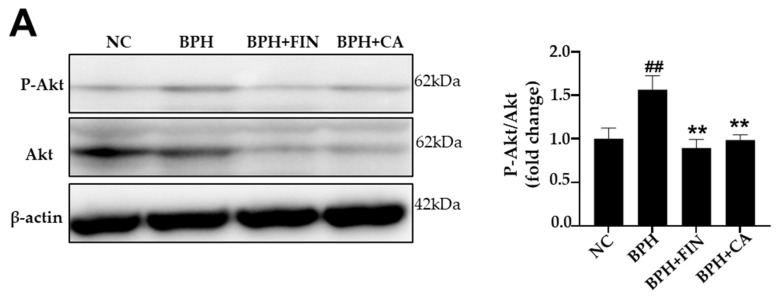

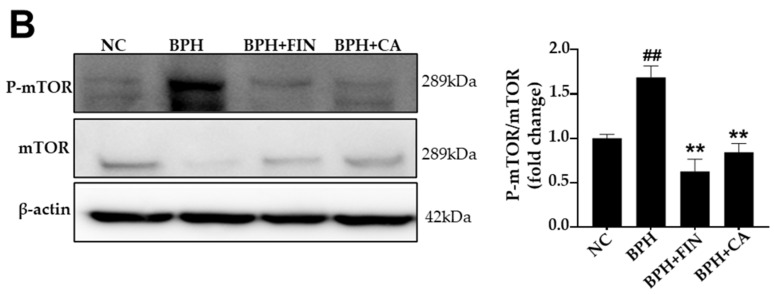
CA reduces the phosphorylation of Akt and mTOR in the rat BPH model. (**A**) The Western blot shows the expression of P-Akt and T-Akt. (**B**) The Western blot shows the expression of P-mTOR and T-mTOR. The NC group consisted of control rats receiving corn oil and PBS. The BPH group injected testosterone propionate (TP, 3 mg/kg) together with PBS. The FIN group received TP in combination with finasteride (10 mg/kg), and the CA group was injected with TP and CA extract (150 mg/kg). Results are reported as mean ± SD. Statistical analysis was conducted using one-way ANOVA, subsequently followed by Tukey’s post hoc test. Statistical significance was defined as ^##^ *p* < 0.01 compared with the NC group, and ** *p* < 0.01 compared with the BPH group.

According to Figure 7A, the phosphorylation of Akt was high in the BPH cohort compared to the NC group, but significantly decreased in the finasteride and CA treatment groups in comparison to the BPH group. Similarly, mTOR phosphorylation was elevated in the BPH group, and this change was reduced by both finasteride and CA treatments (Figure 7B). These results show that CA suppresses the Akt/mTOR signaling pathway in testosterone-induced BPH.

## 3. Discussion

We researched the protective effects of CA extract against the testosterone-stimulated rat BPH model. The results show that CA extract attenuates key markers of the BPH in vivo model, significantly reducing relative prostate weight and serum androgen amounts (testosterone and DHT). Mechanistically, CA extract inhibits the proliferation of prostatic cells, triggers apoptotic signaling, and mitigates the upregulation of growth factors in the rats’ prostates. Taken together, these data suggest that CA extract has a suppressive action on the development of testosterone-induced BPH.

While our findings reveal the significant pharmaceutical capability of the total extract in a BPH model, the individual contribution of each identified constituent was not evaluated separately due to local institutional resource constraints regarding high-purity compound isolation. However, through a systematic literature-based dereplication of our UPLC-QTOF-MS findings, at 280 nm, Tricin-7-O-glucoside is the principal component. This flavone glycoside has been shown to modulate the androgen signaling axis by inhibiting 5-alpha-reductase activity, effectively lowering serum DHT levels [35,36,37,38,39,40,41]. The subsequent reduction in DHT levels attenuates the expression of growth regulators such as TGF-β and IGF-2, thereby halting the over-proliferation of prostatic tissue [28]. Consequently, although Peak 1 (Methyl-flavonoid-pentoside) may contribute to the extract’s overall profile, the high-confidence identification of Tricin-7-O-glucoside (98.67% fit) suggests it is the primary driver of the extract’s ability to restore prostatic homeostasis. However, further research using isolated fractions, Methyl-flavonoid-pentoside and Tri-cin-7-O-glucoside, is required to definitively determine their individual contributions to prostatic proliferation, 5-alpha-reductase activity, and growth factor expression.

Testicular androgens, specifically testosterone and its potent metabolite, DHT, are fundamental regulators of prostate development [11,12]. DHT is primarily synthesized from testosterone via the enzyme 5-alpha-reductase (5-αR). It possesses a high affinity for the AR, and the resulting DHT-AR complex acts as a transcription factor, initiating gene regulation of cellular differentiation and proliferation of prostatic stromal and epithelial cells [4,5]. This process is central to both normal prostate growth and the pathogenesis of BPH. The current pharmacological management strategies against BPH utilize agents such as finasteride, a 4-aza-steroid antagonist of 5-αR. Finasteride selectively inhibits the 5-αR-2 isoenzyme through the formation of high affinity enzyme complex, thereby obstructing the peripheral conversion of testosterone to DHT [11,42]. This action significantly lowers both tissue and serum DHT concentrations, leading to a reduction in overall prostatic volume. Despite its efficacy, however, finasteride is associated with considerable adverse effects, driving the search for substitutes, safer medicinal compounds for BPH management [8,20,43]. In this study, BPH-model rats exhibited elevated serum levels of testosterone and DHT. Crucially, treatment with CA ameliorated these hormonal imbalances and significantly lowered the serum levels of DHT and testosterone.

One of the primary mechanisms underlying BPH is disproportion between cellular apoptosis and proliferation [44,45]. During cell proliferation, PCNA plays a pivotal role in DNA replication and repair [46,47]. In this study, immunohistochemical analysis revealed significantly fewer PCNA-positive nuclei in CA-treated groups in comparison with the BPH group, indicating that CA suppressed cellular proliferation (Figure 4). Moreover, the count of TUNEL-positive cells was markedly elevated in the finasteride and CA-treated groups, indicating that these treatments enhanced apoptotic activity in the rat BPH prostate (Figure 5). Collectively, these results suggest that CA mitigates abnormal prostatic growth in testosterone-induced BPH rats by suppressing cell proliferation and promoting apoptosis.

Growth factors, including VEGF, IGFs, and TGF-β, are central regulators of prostate growth and remodeling in BPH [48,49,50,51]. VEGF, a key mediator of angiogenesis, is expressed in the BPH epithelium and stroma and has been implicated in the stromal proliferation, microvessel formation, and vascular remodeling that accompany prostatic enlargement [50,51]. IGFs (IGF-1 and IGF-2) exert mitogenic effects in the prostate and have been linked to increased prostate size and proliferative signaling in BPH and related disorders [50,51]. TGF-β modulates stromal differentiation, fibroblast activation, and extracellular matrix deposition, thereby contributing to prostatic tissue remodeling in BPH [52]. Consistent with these mechanisms, we found that the mRNA levels of *Vegf*, *Tgf-β*, and *Igf-2* were significantly elevated in prostate tissues from testosterone-treated (BPH) rats versus normal controls. Importantly, administration of CA significantly reduced intraprostatic *Vegf*, *Igf-2*, and *Tgf-β* mRNA expression levels compared to those seen in the untreated BPH group (Figure 6), suggesting that CA modulates pro-growth and pro-angiogenic signaling in this model. Taken together, the present findings suggest that CA extract may attenuate testosterone-induced prostatic enlargement, at least in part, by suppressing the VEGF-, IGF-2-, and TGF-β-mediated pathways that drive angiogenesis, stromal remodeling, and cellular proliferation.

The Akt and mTOR pathways are essential intracellular signaling cascades that regulate cell proliferation, growth, and survival, and are critically involved in the development of BPH [52,53]. Growth regulators such as VEGF, IGF, and TGF-β activate the Akt pathway, leading to the phosphorylation of mTOR and subsequent activation of downstream targets to promote protein synthesis and cell proliferation [54,55]. Excessive activation of Akt and mTOR has been observed in hyperplastic prostate tissue, correlating with increased epithelial proliferation and higher expression of proliferation markers like PCNA [56,57]. Additionally, androgens such as testosterone and DHT can activate Akt phosphorylation through AR-mediated non-genomic signaling to enhance proliferative responses in BPH [58,59,60,61]. We found that the phosphorylation levels of Akt and mTOR were markedly higher in prostate tissues from testosterone-induced BPH rats compared to the normal control group, indicating that the Akt/mTOR pathway was activated in the BPH model rats (Figure 7). Treatment with CA extract markedly decreased Akt phosphorylation, mTOR phosphorylation, and PCNA expression. These actions collectively result in a significant reduction in BPH progression via the targeted blockade of the Akt/mTOR proliferation pathway. These results suggest that CA alleviates prostatic hyperplasia by suppressing P-Akt/P-mTOR to limit abnormal cell growth in our BPH rat model.

## 4. Materials and Methods

### 4.1. Preparation of CA Extract

The *Calamagrostis arundinacea* (L.) Roth (Whole plant) extract (KPM037-065, Code: PB1506.1) was obtained through the Natural Product Central Bank at the Korea Research Institute of Bioscience and Biotechnology (KRIBB, Republic of Korea; https://www.bioone.re.kr/npcb/uss/notice/detail/libraryDetail.do?pageIndex=1&pageUnit=15&searchWrd=&mbId=26&mbSeq=4) (accessed on 19 February 2026). A voucher specimen (KRIB 0029721) is deposited in the KRIBB herbarium. Briefly, 115 g of shade-dried, powdered plant material was added to 1 L of 99.9% methyl alcohol (HPLC grade). Extraction was performed via ultrasonic-assisted extraction using an ultrasonic extractor (SDN-900H, SD-Ultrasonic Co., Ltd., Seoul, Republic of Korea) for 30 cycles (15 min ultrasonication at 40 kHz/1500 W followed by 120 min standing per cycle) at room temperature (25 ± 2 °C). After filtering (Qualitative Filter No. 100 Hyundai Micro Co., Ltd., Seoul, Republic of Korea) the extract was concentrated under decreased pressure using a rotary evaporator (N-1100, EYELA, Tokyo, Japan) at 40 ± 2 °C. Finally, the extract was dried using a freeze-dryer (FD8508, Ilshin Biobased, Dongducheon, Republic of Korea) at −80 ± 5 °C for 72 h to obtain 6.38 g of dry extract. To ensure experimental consistency and minimize variability across all trials, a single standardized batch of this lyophilized CA extract was prepared and used for both the in vitro and in vivo components of the study.

### 4.2. UPLC Analysis

For chromatographic profiling, the CA extract was prepared at a concentration of 1000 ppm with an injection volume of 2 μL. The solution was applied to a Waters ACQUITY™ UPLC I-Class system coupled with a Waters Vion IMS-QTOF-MS. UPLC resolution was achieved on an ACQUITY UPLC^®^ BEH C18 column (1.7 μm, 2.1 × 100 mm) maintained at 35 °C. The consistency of the mobile phase is as follows: 0.1% formic acid in distilled water (Solvent A) and 0.1% formic acid in acetonitrile (Solvent B). The elution was performed at a flow rate of 0.400 mL/min using the following gradient: 0–1 min, 5% B; 20 min, 100% B; 22.3 min, 100% B; 22.4–25 min, 5% B. Mass spectrometry was conducted in both ESI+ and ESI− scan modes with an *m*/*z* range of 100–2000 Da. The desolvation gas (N_2_) flow rate was 800 L/h at a temperature of 350 °C, and the capillary voltage was set to 3000 V.

### 4.3. LNCaP Cell Culture Conditions

Human prostate adenocarcinoma cells (LNCaP), which are androgen-sensitive epithelial cells, were purchased from the American Type Culture Collection (ATCC, Manassas, VA, USA). The cells were maintained in RPMI-1640 medium supplemented with 1% penicillin-streptomycin, 10% fetal bovine serum (FBS), and to ensure optimal growth and stability. The cultures were incubated in a controlled environment containing 5% CO_2_ at 37 °C in a humidified atmosphere. The culture medium was refreshed every 48 to 72 h, and the cells were passaged using 0.25% trypsin-EDTA upon reaching approximately 80% confluence to maintain a logarithmic growth phase for all experimental procedures.

### 4.4. Determination of Cell Viability by MTT Assay

The cytotoxic potential of the CA extract was assessed using the 3-(4,5-dimethylthiazol-2-yl)-2,5-diphenyltetrazolium bromide (MTT) colorimetric assay (Cayman Chemical, Ann Arbor, MI, USA, and ALPCO Diagnostics, Salem, NH, USA, respectively). LNCaP cells were seeded in 96-well plates at a density of 1 × 10^4^ cells per well and incubated to adhere for 24 h. Subsequently, after that cells were treated with different concentrations of the CA extract (50, 100, and 200 µg/mL) or a vehicle control (0.1% DMSO in culture medium) for 24 h. Following incubation, 20 µL of MTT solution (5 mg/mL) was added to each well and incubated for 4 h at 37 °C to allow for the formation of purple formazan crystals by viable mitochondrial dehydrogenases. The supernatant was then aspirated, and 150 µL of dimethyl sulfoxide (DMSO) was added to dissolve the formazan. A microplate reader was used to determine the optical density (OD) at a wavelength of 570 nm. Percent cell viability was determined relative to the vehicle-treated control group, providing evidence of the extract’s safety profile at the tested concentrations.

### 4.5. Animals

7 weeks old Male Sprague–Dawley rats were bought from Orient Bio (Seong-nam, Republic of Korea). Rats were kept under control conditions (22 ± 2 °C; 50 ± 5% relative humidity; 12 h light/dark cycle) with ad libitum access to standard chow and sterilized water. The Institutional Animal Care and Use Committee (IACUC) of Chungnam National University (Daejeon, Republic of Korea) approved all experimental procedures.

### 4.6. Induction of BPH and Treatment Protocol

BPH was caused in Sprague–Dawley male rats by everyday subcutaneous administration of testosterone propionate (TP; 3 mg/kg; Tokyo Chemical Industry Co., Tokoyo, Japan) for 4 consecutive weeks. All treatments were given once daily throughout the experimental period. Rats were randomly sorted to 4 experimental cohorts (*n* = 8 representative rats per group): the NC group received phosphate-buffered saline (PBS) orally and corn oil subcutaneously as vehicles; the BPH group received TP subcutaneously and PBS orally; the finasteride-treated group (BPH + FIN, positive control) received TP plus oral finasteride (10 mg/kg; Sigma, St. Louis, MO, USA); and CA-treated group (BPH + CA) received TP plus oral administration of CA extract at 150 mg/kg. The dose of 150 mg/kg was selected in accordance with effective doses for other Poaceae family extracts in similar rat models [62,63,64]. The oral administration volume was maintained at 5 mL/kg, and subcutaneous injections were given at 3 mL/kg. Body weights were recorded weekly and on the necropsy day. After the final administration, rats were anesthetized and euthanized, blood samples were collected from the vena cava, and prostates were excised, weighed, and processed for biochemical and histological analyses.

### 4.7. Quantification of Serum Testosterone and DHT

Testosterone and DHT levels within the serum were quantified using commercial ELI-SA kits (Cayman Chemical, Ann Arbor, MI, USA, and ALPCO Diagnostics, Salem, NH, USA, respectively) following the manufacturers’ protocol. Serum samples were incubated with enzyme conjugates and washed to remove unbound reagents, and substrate was added. Absorbance was measured at 412 nm (testosterone) and 450 nm (DHT) using a microplate reader, and concentrations were calculated from standard curves.

### 4.8. Histological Analysis

Prostate tissues were fixed in 10% neutral-buffered formalin, embedded in paraffin, and sectioned at 4-μm thickness. Sections were stained with hematoxylin and eosin (H&E). Epithelial thickness of the ventral prostate was quantified in 5 arbitrarily selected fields (400× magnification) per rat (*n* = 8 representative rats per group) using the software of ImageJ (version 46a; Bethesda, NIH, MD, USA) as described previously [24].

### 4.9. Immunohistochemistry (IHC) for PCNA

Paraffin-embedded sections were deparaffinized in xylene and rehydrated through graded ethanol (100%, 95%, 90%, 80%, 70% and 50% *v*/*v* for 1 min and 30 s each). After heat-induced epitope retrieval, the sections were blocked with normal goat serum (Vectastain, Cambridge, UK) and incubated for 12 h at 4 ± 1 °C with a primary antibody against proliferating cell nuclear antigen (PCNA; Abcam, Cambridge, UK; dilution 1:10,000) [36,37]. The sections were then treated with a biotinylated secondary antibody (Vectastain), and the results were visualized using a diaminobenzidine (DAB; Vector Laboratories, Newark, CA, USA) substrate. Five random high-power fields (400×) per sample (*n* = 8 representative rats per group) were analyzed, and PCNA-positive cells were expressed as the ratio of positive nuclei per 100 cells.

### 4.10. TUNEL Assay

Apoptotic cells were detected with a commercial detection TUNEL kit (Merck Millipore Corporation, Burlington, MA, USA) according to the protocol provided by the manufacturer. Tissues were deparaffinized, rehydrated, and treated with proteinase K, and endogenous peroxidase was inactivated with 3% hydrogen peroxide (Samchun Chemical Co., Seoul, Republic of Korea). DNA fragmentation was labeled with biotinylated dUTP in the presence of terminal deoxynucleotidyl transferase (TdT). Signal detection was performed using DAB, and nuclei were counterstained with hematoxylin. Five random fields (400×) per section (*n* = 8 representative rats per group) were examined, and TUNEL-positive nuclei were counted and expressed as the ratio of positive cells per 100 cells. For TUNEL experiments, five fields per rat were randomly selected to ensure statistical representation.

### 4.11. Western Blot Analysis

Prostate tissues were subjected to homogenization in RIPA lysis buffer (Cell Signaling Technology, Danvers, MA, USA), and total proteins were obtained. Equal amounts of protein (15 µg) were separated by SDS-PAGE alongside molecular weight markers (10–250 kDa) to confirm protein identity and transferred onto PVDF membranes. Membranes were blocked and incubated for 12 h at 4 ± 1 °C with the following primary antibodies: anti-Akt, anti-phospho-Akt, anti-mTOR, anti-phospho-mTOR, and anti-β-actin (Cell Signaling Technology). This was followed by incubation with HRP-conjugated secondary antibodies for 2 h at 25 ± 2 °C, and protein bands were detected using an enhanced chemiluminescence detection kit. Densitometric analysis was performed using the CS Analyzer 4 software (Atto, Tokyo, Japan).

### 4.12. Quantitative Real-Time PCR (RT-qPCR)

Total RNA was harvested from prostate tissue using the TRIzol reagent (Thermo Scientific, Waltham, MA, USA). cDNA synthesis was performed with 1.4 µg of total RNA using a commercial RT kit (Toyobo, Osaka, Japan). RT-qPCR was conducted with SYBR Green Master Mix (Thermo Scientific). The primer sequences for *Tgf-β1*, *Igf-2*, *Vegf*, and *Gapdh* were as follows: *Igf-2*, f:5′-GTCTGTGCCTCAGCCTCTTC-3′ and r:5′-CCCATTTGGGAACTTCGCCT-3′; *Tgf-β*, f:5′-AGGGCTCAACACCTGCAC-3′ and r:5′-GGGCCCCAGACAGAAGTT-3′; *Vegf*, f:5′-GAGGAAAGGGAAAGGGTCAAAA-3′ and r: 5′-CACAGTGAACGCTCCAGGATT-3′; *Gapdh* f:5′-CAACTCCCTCAAGATTGTCAGCAA-3′ and r:5-‘GGCATGGACTGTGGTCATGA-3′. Comparative 2^−ΔΔCt^ method was applied to determine relative mRNA expression levels, with *Gapdh* serving as the internal reference control for normalization.

### 4.13. Statistical Assessment

Results are reported as mean ± SD. Statistical significance among groups was evaluated by one-way analysis of variance (ANOVA) paired by Tukey’s multiple comparison test, using the GraphPad Prism 9 software (GraphPad Software, Boston, CA, USA). *p* < 0.05 was considered statistically significant.

## 5. Conclusions

In summary, this research demonstrates that CA extract effectively inhibits the development of testosterone-triggered BPH in rats, reducing prostate weight and epithelial hyperplasia. These protective effects are mediated at least partly through the downregulation of serum testosterone and DHT. CA also exhibits anti-proliferative, pro-apoptotic, and anti-growth-factor activities, suggesting that it may help restore the balance between cell proliferation and apoptosis in prostatic tissue. Collectively, our results indicate that CA may serve as a viable natural candidate for the prevention and management of BPH.

## Figures and Tables

**Table 1:** CA extract effect on relative prostate weight indexes and reduction in growth.

Group	Treatment	Mass of Body (g)	Relative Prostate Mass (%)	Growth Reduction (%)
NC	PBS + Corn oil	431.2 ± 19.1	0.186 ± 0.032	
BPH	PBS + TP	393.1 ± 14.4	0.345 ± 0.076 **^##^**	
BPH + FIN	Finasteride + TP	450.1 ± 24.5	0.215 ± 0.059 *	37.86
BPH + CA	CA150 mg/kg + TP	463.1 ± 23.2	0.286 ± 0.045 *	17.34

Effects of CA extract on prostate relative weight indices and reduction in prostatic enlargement. The NC group consisted of control rats receiving corn oil and PBS. The BPH group injected testosterone propionate (TP, 3 mg/kg) together with PBS. The FIN group received TP in combination with finasteride (10 mg/kg), and the CA group was injected with TP and CA extract (150 mg/kg). Results are reported as mean ± SD. Statistical analysis was conducted using one-way ANOVA, subsequently followed by Tukey’s post hoc test. Statistical significance was defined as ^##^ *p* < 0.01 compared with the NC group, and * *p* < 0.05 compared with the BPH group.

## Data Availability

The original contributions presented in this study are included in the article. Further inquiries can be directed to the corresponding authors.

## Abbreviations

5-AR, 5α-reductase; 5-ARIs, 5α-reductase inhibitors; α1-blockers, alpha1-adrenergic antagonists; AKT, protein kinase B; p-AKT, phosphorylated protein kinase B; AR, androgen receptor; BPH, benign prostatic hyperplasia; CA, *Calamagrostis arundinacea*; DHT, dihydrotestosterone; FGF, fibroblast growth factor; H&E, hematoxylin and eosin; IGFs, insulin-like growth factors; LUTS, lower urinary tract symptoms; mTOR, mammalian target of rapamycin; p-mTOR, phosphorylated mammalian target of rapamycin; PCNA, proliferating cell nuclear antigen; PDE5Is, phosphodiesterase 5 inhibitors; PSA, prostate-specific antigen; SC, subcutaneous; TGF-β, transforming growth factor beta; TP, testosterone propionate; TUNEL, terminal deoxynucleotidyl transferase dUTP nick-end labeling; VEGF, vascular endothelial growth factor.
